# New insights into family relationships within the avian superfamily Sylvioidea (Passeriformes) based on seven molecular markers

**DOI:** 10.1186/1471-2148-12-157

**Published:** 2012-08-25

**Authors:** Silke Fregin, Martin Haase, Urban Olsson, Per Alström

**Affiliations:** 1 Vogelwarte Hiddensee, Zoological Institute and Museum, Ernst Moritz Arndt University of Greifswald, Greifswald, 17489, Germany; 2 Department of Zoology, Systematics and Biodiversity, University of Gothenburg, Box 463, Göteborg, SE-405 30, Sweden; 3 Key Laboratory of Zoological Systematics and Evolution, Institute of Zoology, Chinese Academy of Sciences, 1 Beichen West Road, Beijing, Chaoyang District, 100101, Peoples Republic of China; 4 Swedish Species Information Centre, Swedish University of Agricultural Sciences, Box 7007, Uppsala, SE-750 07, Sweden

**Keywords:** Phylogeny, Passerines, Taxonomic revision, International Code of Zoological Nomenclature

## Abstract

**Background:**

The circumscription of the avian superfamily Sylvioidea is a matter of long ongoing debate. While the overall inclusiveness has now been mostly agreed on and 20 families recognised, the phylogenetic relationships among the families are largely unknown. We here present a phylogenetic hypothesis for Sylvioidea based on one mitochondrial and six nuclear markers, in total ~6.3 kbp, for 79 ingroup species representing all currently recognised families and some species with uncertain affinities, making this the most comprehensive analysis of this taxon.

**Results:**

The resolution, especially of the deeper nodes, is much improved compared to previous studies. However, many relationships among families remain uncertain and are in need of verification. Most families themselves are very well supported based on the total data set and also by indels. Our data do not support the inclusion of *Hylia* in Cettiidae, but do not strongly reject a close relationship with Cettiidae either. The genera *Scotocerca* and *Erythrocercus* are closely related to Cettiidae, but separated by relatively long internodes. The families Paridae, Remizidae and Stenostiridae clustered among the outgroup taxa and not within Sylvioidea.

**Conclusions:**

Although the phylogenetic position of *Hylia* is uncertain, we tentatively support the recognition of the family Hyliidae Bannerman, 1923 for this genus and *Pholidornis*. We propose new family names for the genera *Scotocerca* and *Erythrocercus*, Scotocercidae and Erythrocercidae, respectively, rather than including these in Cettiidae, and we formally propose the name Macrosphenidae, which has been in informal use for some time. We recommend that Paridae, Remizidae and Stenostiridae are not included in Sylvioidea. We also briefly discuss the problems of providing a morphological diagnosis when proposing a new family-group name (or genus-group name) based on a clade.

## Background

The order Passeriformes, also called passerines or perching-birds, is the largest of the 40 orders within the class Aves, including ~60% of all ~10500 living bird species [1]. The passerines are divided into three major groups, with Acanthisittidae (New Zealand wrens) being sister to the two large parvorders oscines and suboscines [2-5]. Oscines, “true” songbirds, possess a complex syrinx, which enables them to perform complex songs, whereas suboscines do not have this characteristic [6,7]. Passerida, the largest group within oscines, can only be delimited by an insertion of one amino acid in exon 3 of the c-myc gene [8], but no synapomorphic morphological character is known to define this taxon. Within Passerida, the superfamily Sylvioidea has proved difficult to delineate based on morphology, because of apparent multiple events of convergent evolution [e.g. [9-12]. Several of these studies found evidence that Sylvioidea *sensu* Sibley and Ahlquist [12] and Sibley and Monroe [13], which was based on DNA-DNA hybridization studies, was not monophyletic. Recently, Sylvioidea has gone through a profound rearrangement based on various sets of molecular sequence data [14-18]. These studies showed that several of the families and subfamilies established by Sibley and Ahlquist [12] were non-monophyletic.

The first comprehensive study of the whole superfamily, by Alström et al. [14], was based on one nuclear and one mtDNA sequence. That study identified 10 well supported major clades, which were proposed to be recognized at the family level. One of the consequences of that revision was a temporary loss of the family name Sylviidae, which was previously recognized as the largest family within Sylvioidea. As the type genus of Sylviidae Leach, 1820, *Sylvia*, was shown to be nested within the large Timaliidae Vigors and Horsfield, 1827 assemblage, it was suggested to suppress Sylviidae, following the principle of stability [9,14,19]. However, Sylviidae was re-established by Gelang et al. [17], to coexist as a separate family along with Timaliidae.

Following the above changes, Sylvioidea comprised 20 families containing in total more than 1200 species in 221 genera. Table 1 shows the latest printed classification by Dickinson [20] and the continuously updated IOC World Bird List [1]. The latter classification has taken all of the recent molecular advances into account. The most recent changes were that the monotypic genera *Panurus* and *Nicator* were raised to family level, Panuridae and Nicatoridae, respectively (cf. [11,14,16,18]; Macrosphenidae was used as family-name for the “*Sphenoeacus* group” (cf. [16,18]; the name Megaluridae was synonymized with Locustellidae, as the latter was found to have priority [21]; the family Pnoepygidae was proposed for the genus *Pnoepyga*[17]; the four subfamilies Timaliinae, Pellorneinae, Leiotrichinae and Zosteropinae recognized within Timaliidae [17] were all elevated to family rank; and *ScotocercaErythrocercus* and *Hylia* were tentatively included in Cettiidae (cf. [16,18,22-26].

**Table 1:** Families within Sylvioidea, and genera included in present study

**Dickinson (2003)[**[20]**]**		**Gill and Donsker (2011)[**[1]**]**	
		^**1**^**Panuridae**	*Panurus*
		^**2**^**Nicatoridae**	*Nicator*
		^**3**^**Alaudidae**	*Mirafra, Ammomanes, Alauda*
		^**4**^**Pycnonotidae**	*Pycnonotus, Arizelocichla (Andropadus), Atimastillas (Chlorocichla), Phyllastrephus, Hypsipetes (Ixos)*
**Hirundinidae**	Hirundininae*: Hirundo*^5^, *Delichon*^5^	^**5**^**Hirundinidae**	*Hirundo, Delichon*
		^**6**^**Pnoepygidae**	*Pnoepyga*
		^**7**^**Macrosphenidae**	*Melocichla, Sphenoeacus, Macrosphenus, Sylvietta, Cryptillas (Bradypterus)*
		^**8**^**Cettiidae**	*Scotocerca, Erythrocercus, Tesia, Cettia, Abroscopus, Hylia*
**Aegithalidae**	*Aegithalos*^9^, *Leptopoecile*^9^, *Psaltriparus*^9^	^**9**^**Aegithalidae**	*Aegithalos, Leptopoecile, Psaltiparus*
		^**10**^**Phylloscopidae**	*Phylloscopus, Seicercus*
		^**11**^**Acrocephalidae**	*Nesillas, Acrocephalus, Calamonastides (Chloropeta), Hippolais*
		^**12**^**Locustellidae**	*Dromaeocercus, Megalurus, Bradypterus, Locustella*
		^**13**^**Donacobiidae**	*Donacobius*
		^**14**^**Bernieridae**	*Oxylabes, Bernieria, Hartertula, Thamnornis, Xanthomixis, Crossleyia*
**Alaudidae**	*Mirafra*^3^, *Ammomanes*^3^, *Alauda*^3^		
**Cisticolidae**	*Cisticola*^15^, *Scotocerca*^8^, *Prinia*^15^, *Spiloptila*^15^, *Apalis*^15^, *Hypergerus*^15^, *Camaroptera*^15^, *Calamonastes*^15^	^**15**^**Cisticolidae**	*Cisticola, Prinia, Spiloptila, Apalis, Hypergerus, Camaroptera, Calamonastes, Orthotomus, Artisornis, Eremomela*
**Genera incertae sedis**	*Orthotomus*^15^, *Artisornis*^15^		
**Pycnonotidae**	*Pycnonotus*^4^, *Andropadus*^4^, *Chlorocichla*^4^, *Phyllastrephus*^4^, *Ixos*^4^		
**Genera incertae sedis**	*Nicator*^2^, *Erythrocercus*^8^		
**Sylviidae**	Megalurinae*: Megalurus*^12^; Acrocephalinae: *Tesia*^8,^*Cettia*^8^, *Bradypterus*^7,12^, *Dromaeocercus*^12^, *Nesillas*^11^, *Melocichla*^7^, *Sphenoeacus*^7^, *Locustella*^12^, *Acrocephalus*^11^, *Hippolais*^11^		
**Genera incertae sedis**	*Macrosphenus*^7^, *Hylia*^8^, *Oxylabes*^14^, *Bernieria*^14^, *Hartertula*^14^, *Thamnornis*^14^, *Xanthomixis*^14^, *Crossleyia*^14^		
	Phylloscopinae*: Phylloscopus*^10^, *Seicercus*^10^, *Abroscopus*^8^, *Eremomela*^15^, *Sylvietta*^7^, Sylviinae*: Sylvia*^19^		
**Timaliidae**	*Pellorneum*^17^, *Illadopsis*^17^, *Pseudoalcippe*^19^, *Pnoepyga*^6^*,Stachyris*^16^, *Dumetia*^16^, *Chrysomma*^19^, *Chamaea*^19^, *Turdoides*^18^, *Garrulax*^18^, *Alcippe*^19^, *Phyllanthus*^18^, *Yuhina*^20^, Erpornis^*^, *Panurus*^1^, *Paradoxornis*^19^	^**16**^**Timaliidae**	*Stachyris, Dumetia*
		^**17**^**Pellorneidae**	*Illadopsis, Pellorneum*
		^**18**^**Leiothrichidae**	*Phyllanthus, Turdoides, Trochalopteron (Garrulax)*
		^**19**^**Sylviidae**	*Pseudoalcippe, Sylvia, Lioparus, Chrysomma, Chamaea, Sinosuthora (Paradoxornis)*
**Genera incertae sedis**	Chaetops^*^		
**Zosteropidae**	*Zosterops*^20^	^**20**^**Zosteropidae**	*Yuhina, Zosterops*

Superscript numbers in second column refer to numbers in third column, indicating new family affiliations. Genera in brackets give the name used by Dickinson [20], which have changed according to Gill and Donsker [1]. *No longer included in Sylvioidea.

Despite the numerous studies on large-scale relationships within Sylvioidea, the relationships among the families are still largely unresolved. We here present a multilocus analysis of one mitochondrial and six nuclear markers, ~6300 aligned basepairs for 79 species with the aim to clarify the phylogeny.

## Results

### Sequence statistics

The combined dataset comprised 6332 aligned basepairs of nucleotide sequence data, one mitochondrial and six nuclear markers. Percentage of parsimony informative sites were as follows: recombination activating gene 1 (RAG1) 34% (652/1934), fibrinogen beta chain (FGB) 36% (229/632), glyceraldehyde-3-phosphate dehydrogenase (GAPDH) 38% (166/439), myoglobin (MB) 42% (319/765), ornithine decarboxylase 1 (ODC1) 45% (355/796), mtDNA cytochrome b (MT-CYB) 46% (531/1143), and lactate dehydrogenase B (LDHB) 47% (291/624).

GARLI-PART found the tree with the highest likelihood in 53 of all 100 runs, the next best tree was found in 27 of the runs. These trees differed only in the topology of the outgroup taxa. Thus, in 80 out of 100 inferences, GARLI-PART found the same topology within Sylvioidea, which was identical to the Bayesian inference (BI) 50% majority rule tree with respect to the relationships within Sylvioidea.

In the BI, 80/78% (combined/nuclear data) of the nodes were well supported (PP ≥0.95), 17/17% had PPs between 0.51 and 0.94, and only 2/5% of the nodes were unresolved. In the ML analyses, 61/50% of the nodes had support values ≥85%, 26/28% between 50% and 84%, and 13/22% <50%.

### Phylogeny of Sylvioidea

**Figure 1:**
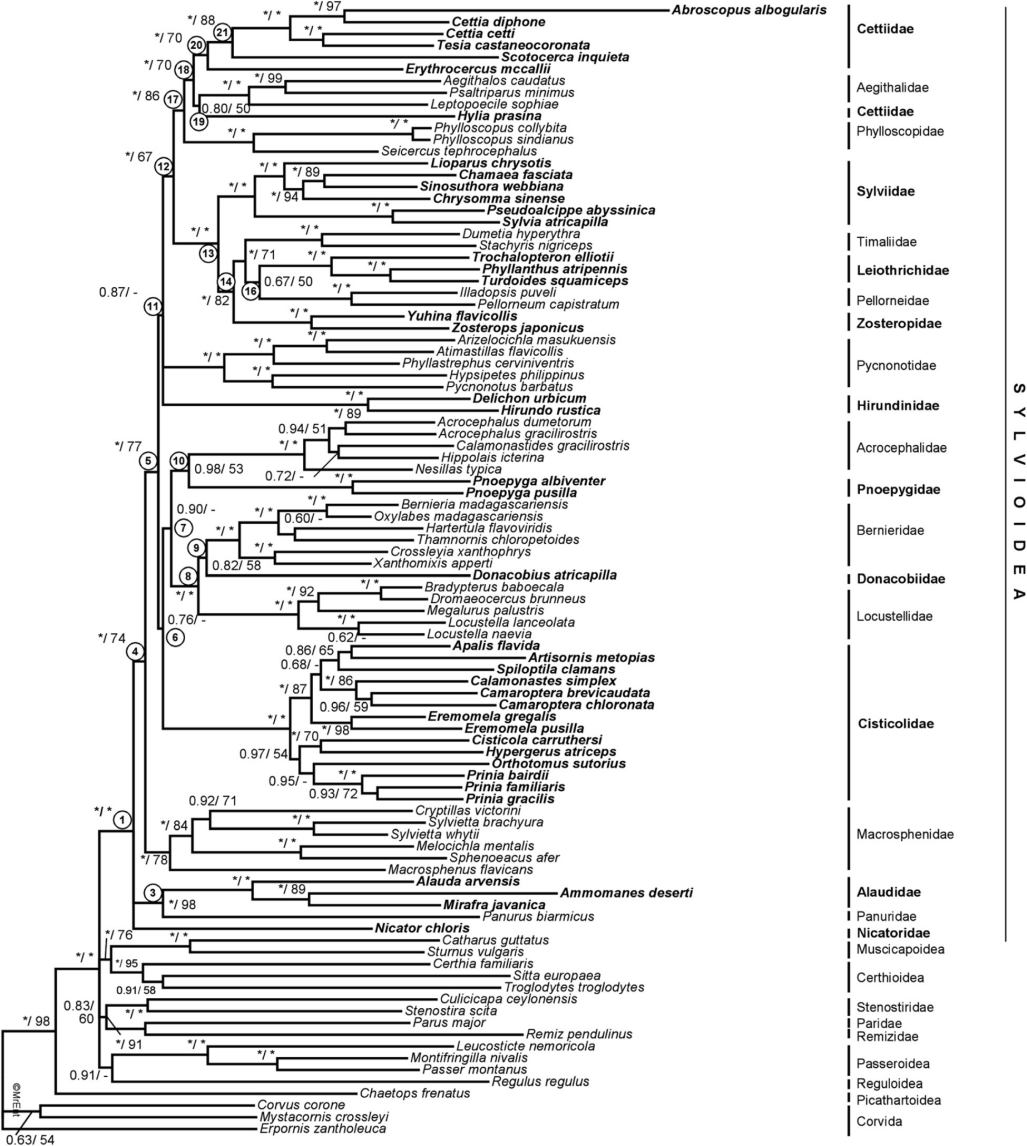
**Phylogeny of Sylvioidea based on the complete data set.** Phylogenetic tree based on the complete concatenated dataset (MT-CYB, FGB, GAPDH, LDHB, MB, ODC1, RAG1), analysed by Bayesian inference (partitioned by locus). Support values are given in the order posterior probability (PP) / maximum likelihood (ML) bootstrap; * indicates PP 1.00 or ML 100%; - indicates no ML bootstrap support for this node, but clade recovered in ML search for best topology. For better clarity, families belonging to Sylvioidea are alternately written in bold. Node numbers are the same as in Figure 2.

**Figure 2:**
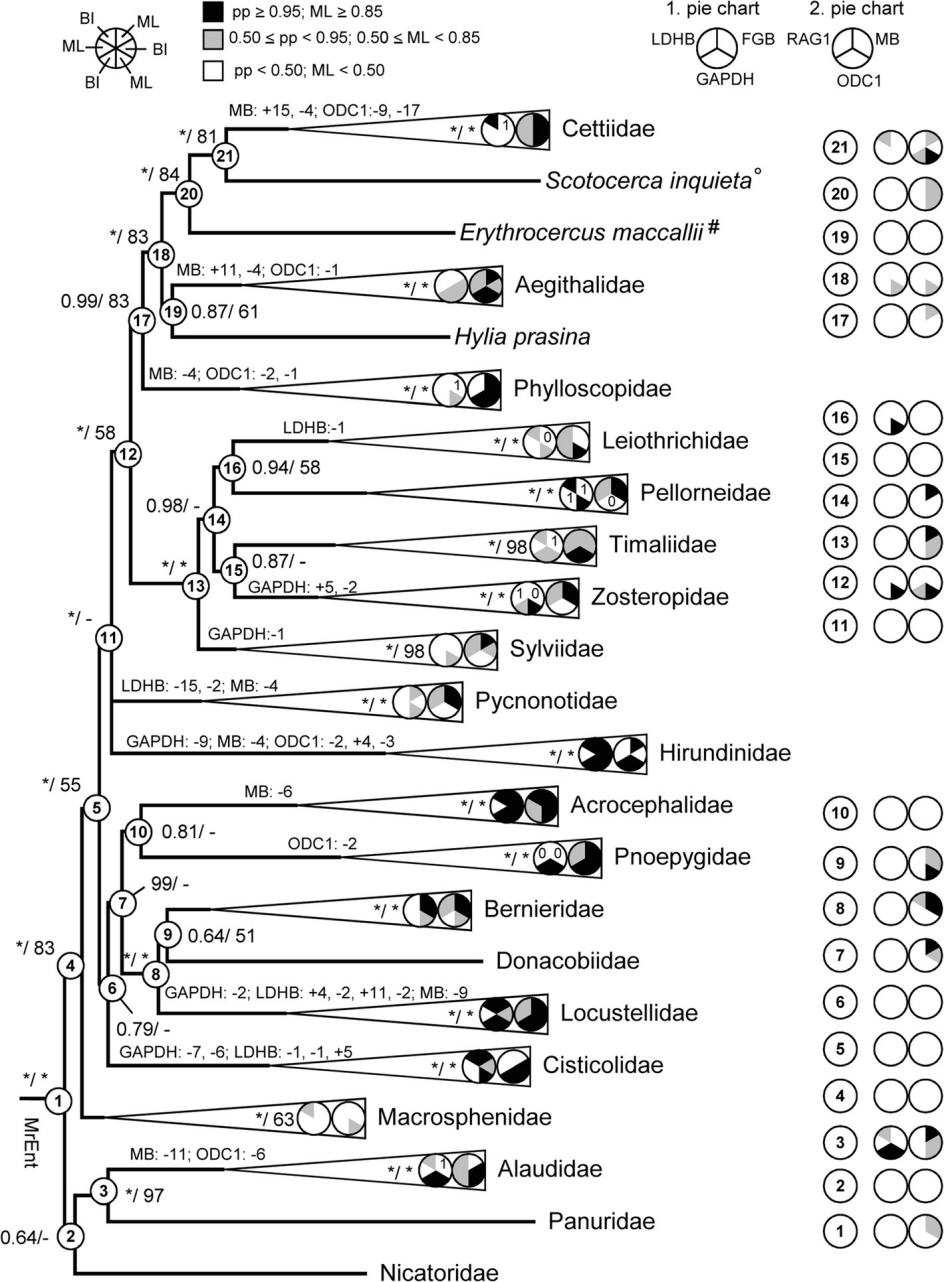
**Phylogeny of Sylvioidea based on the nuclear data set.** Phylogenetic tree based on the nuclear data set (FGB, GAPDH, LDHB, MB, ODC1, RAG1), analysed by Bayesian inference (partitioned by locus). Support values are given in the order posterior probability (PP) / maximum likelihood (ML) bootstrap; * indicates PP 1.00 or ML 100%; - indicates no ML bootstrap support, but clade recovered in ML search for best topology. Pie charts indicate support in the six nuclear single-locus analyses, first pie chart refers to FGB, GAPDH and LDHB; second pie chart refers to MB, ODC1 and RAG1. Pie charts within family clades indicate support for the family itself, whereas pie charts on the right show support for nodes indicated on the tree. Numbers in pie charts indicate if no (0) or only one (1) sequence of the respective genetic marker was available. #: no LDHB or RAG1 available, °: no FGB or RAG1 available. Indels supporting individual families are given on the respective branches.

The tree based on the complete dataset is shown in Figure 1, and the tree based on the nuclear dataset is shown in Figure 2, with the results from the single-locus analyses indicated in the latter figure. There is generally good agreement between these two trees. The same applies to the analysis in 14 partitions, which recovered basically the same topology with similar nodal support, and with no well supported conflicts. All families in Sylvioidea (excluding monotypic families) had PP 1.00 and ML bootstrap support 100%, except Macrosphenidae and Cettiidae *sensu* Gill and Donsker [1] (Macrosphenidae had PP 1.00 and ML bootstrap 78%; Cettiidae *sensu* Alström et al. [14] had 1.00/100% support).

Nicatoridae, Alaudidae and Panuridae were sister to all other sylvioid taxa (node 4), with PP 1.00 but lower ML bootstrap support. The sister relationship of Alaudidae and Panuridae was highly supported in the combined and nuclear analyses. Macrosphenidae was sister to the other sylvioid families (node 5), albeit less supported in the ML bootstrap analyses of the combined data set.

The remaining families were divided into two major clades, 6 and 11. Clade 6 consisted of Cisticolidae, Locustellidae, Bernieridae, Donacobiidae, Acrocephalidae, and Pnoepygidae. These relationships were mostly only supported by BI, although clade 8, containing Bernieridae, Donacobiidae and Locustellidae, was strongly supported by both BI and ML. The sister relationship of Donacobiidae and Bernieridae (node 9) was weakly supported in all analyses. The sister clade to Cisticolidae (7) had varying support in the combined and nuclear analyses.

The largest clade (11) was poorly supported, with a basal polytomy consisting of Hirundinidae, Pycnonotidae and a clade (12) containing the remaining families. Within clade 12, the strongly supported clade 13 comprised Zosteropidae, Timaliidae, Pellorneidae, and Leiothrichidae with Sylviidae as their common sister group. The relationships among the families in clade 14 were uncertain, and differed between the analysis of the complete dataset and the one based on only nuclear loci. The sister relationship between Leiothrichidae and Pellorneidae, only weakly supported in the combined data set, was well (PP 0.94) supported in the nuclear data set, but not well by ML.

Clade 17 formed the sister clade to the sylviid/timaliid taxa (13), although the clade (12) containing these two clades received low ML bootstrap support. Within clade 17, Phylloscopidae was sister to a clade (18) containing Aegithalidae and a non-monophyletic Cettiidae. The sister relationship of Aegithalidae and the cettiid genus *Hylia* was poorly supported. The clade containing *Erythrocercus*, *Scotocerca* and other Cettiidae (20) was well supported, especially by the nuclear data set, as was the *Scotocerca*/other Cettiidae clade (21).

There were only few strongly supported incongruences: 1) the sister relationship of *Ammomanes deserti* and *Mirafra javanica* (in Alaudidae) found by the complete and nuclear data sets, was strongly contradicted (PP 0.92–1.00) by the single-locus analyses of MB, GAPDH and MT-CYB, which instead supported a sister relationship of *Alauda arvensis* and *Mirafra javanica*. 2) *Sinosuthora webbiana* was placed in Pellorneidae by FGB (PP 1.00). 3) *Donacobius* was sister to Locustellidae based on FGB, but sister to Bernieridae using ODC1. 4) *Trochalopteron elliotii* was placed in Pellorneidae and not in Leiothrichidae in the GAPDH tree (PP 1.00).

### Indels

Most families had unique insertions and/or deletions (indels), which lent further support to these clades (Figure 2). However, few indels were shared by two or more families (Figure 2). The grouping of Panuridae with Alaudidae was supported by an insertion of 6 bp in ODC1. *Erythrocercus* and *Scotocerca* shared a 9 bp deletion in ODC1 with the other Cettiidae, except *Hylia*. A 4 bp deletion in MB was shared by the taxa in clade 17 (Phylloscopidae, Aegithalidae and Cettiidae), but this was also found in Pycnonotidae and Hirundinidae, which were inferred to be more distantly related. Two deletions of three basepairs in FGB and MB, respectively, delimited Sylvioidea from the outgroup, including Paridae, Remizidae and Stenostiridae. The inclusion of *Eremomela* in Cisticolidae was supported by several shared indels.

## Discussion

### Phylogeny of Sylvioidea

The present study is the most comprehensive analysis of the superfamily Sylvioidea, both with respect to the number of taxa and the number of loci. BI and ML searches found identical topologies, which reinforces the confidence in the results, even though the strength of the support differed between these methods. Only few deeper nodes (except those defining families) were supported by single-locus analyses. MB and ODC1 provided most resolution deep in the tree, and MB was the only single marker that supported Sylvioidea as a monophyletic group in the BI and ML bootstrap. The best ML trees for FGB and RAG1 also inferred Sylvioidea to be monophyletic, but this was not supported by their respective bootstrap analyses. Thus, the concatenation of all markers improved the resolution substantially.

The overall support of the multilocus tree, especially of the deeper nodes, is much improved compared to previous studies [14,16,18]. Especially studies using only mitochondrial data have failed to resolve most nodes below family level [27-29]. However, also an analysis by Johansson et al. [18] of a dataset comprising six loci (MB, ODC1, FGB, RAG1, RAG2 and ND2; in total ~7.3 kbp) for 14 sylvioid taxa was largely unresolved. The short internodes and lack of resolution deep in the tree suggest a rapid radiation of the families within Sylvioidea.

The sister relationship of Alaudidae and Panuridae, which is extremely unexpected from a morphological and ecological perspective, was very well supported, also by several single-locus analyses. This relationship has been found also in previous studies based on fewer, but partly the same, loci [11,14,18,23]. The precise position of the enigmatic Nicatoridae still has to be regarded as uncertain.

The position of Macrosphenidae as sister to the remaining sylvioid taxa was well supported in the BI but less so in the ML bootstrap analyses. This has previously been found based on different taxon samplings and partly different loci [16,18,21]. In contrast, in studies where only one mitochondrial and one nuclear loci were used [14,23] Macrosphenidae was placed in a more derived position within Sylvioidea.

The two large clades 6 and 11 have been inferred in two previous studies based on different taxon sampling and some of the same loci as in the present analysis [21,22], although they have not been recovered in other studies based on different taxon sampling and partly different loci [16,18]. As they were poorly supported here, they are to be considered as highly tentative.

Clade 7 in general was also found by various studies, but with differing constellations. While clade 8 was quite consistently recovered in previous studies [17,18,21,22], as well as in studies lacking either Donacobiidae or Bernieridae [14,16], the relationships among clade 8, Acrocephalidae and Pnoepygidae varied. The latter family was found as sister to clade 8 and Acrocephalidae [22,24] or in different positions [17], though never well supported. Lei et al. [27] found in a study based solely on mitochondrial sequences a close relationship between Locustellidae and Cisticolidae, but with Acrocephalidae falling in another clade, with high support in the Bayesian analysis, but with only low ML bootstrap support.

The largest clade (11) was divided into a polytomy formed by Pycnonotidae, Hirundinidae and clade 12. Pycnonotidae, Hirundinidae and clade 17 shared a 4 bp deletion in MB that was not found in clade 13. Due to the somewhat uncertain relationships in the deeper nodes in this part of the tree, different scenarios are possible. One is that this deletion was reversed by the members of clade 13, or that the different families lost these base pairs independently. Alternatively, the homoplastic appearance of this indel could be a case of hemiplasy [30], where the gene tree is not congruent with the species tree due to lineage sorting. Hemiplasy is considered to be more likely when internodes are short [30,31], as is the case in this clade. In a study of transposable elements over a wide range of birds, cases of homoplasy were found, but lineage sorting was considered an unlikely explanation of these events [32]. However, indels seem to be more prone to homoplasy than insertions of transposable elements [cf. [31,33].

Clade 12 was recovered also by Johansson et al. [18] (their Figure 2, clade I). Within clade 12, clade 13 consisted of the much debated sylviid/timaliid families. All these families had very high support in our study, as well as the whole clade itself (13), whereas the latter was only weakly supported in the ML analysis in Gelang et al. [17]. The relationships among the families in clade 13 agreed with Gelang et al. [17], although they were better supported in the latter study, which was based on a much denser taxon sampling but fewer loci than the present study. Sylviidae, when studied in larger sample sizes together with former Timaliidae and based on more than one locus [9,17,24], was always found as a separate clade. Gelang et al. [17] recognised Leiothrichinae, Pellorneinae, Timaliinae and Zosteropinae as subfamilies within Timaliidae, whereas Gill and Donsker [1] elevated these to family rank. We support the latter treatment, as it is more on a par with the treatment of the other groups within Sylvioidea.

The close affinities of Phylloscopidae, Aegithalidae and Cettiidae (clade 17) were well supported by our nuclear data set, although the relationships among these are not unanimously well supported by both BI and ML. This clade had been found previously [14,18,21,22], although with weaker support. The latter study [22] also noted morphological similarities between Cettiidae *sensu* Alström et al. [14], *ScotocercaErythrocercus* and Aegithalidae, especially between the first two ( *Hylia* not examined).

The families Paridae, Remizidae and Stenostiridae are sometimes included in Sylvioidea [e.g. 13 (excluding Stenostiridae), [34-36]. Based on the phylogeny presented here, additional evidence from indels, and previous studies, we recommend that these three families are not included in Sylvioidea, and accordingly that Sylvioidea is circumscribed as in Figures 1 and 2.

### Intrafamilial relationships

Macrosphenidae was the least supported family within Sylvioidea, and none of the single-locus analyses recovered this group with high support. This is probably the result of long divergence times among the different species or species pairs included here, as indicated by long branches. This clade contains species that are morphologically and ecologically highly divergent, and this in combination with some long internodes within this clade suggest that a number of extant and/or extinct taxa also belong here. In addition to the genera included here, also *Achaetops* has been shown to belong in this group [16].

Our results confirm the general structure within Cisticolidae recovered by Nguembock et al. [37]. We also corroborate the sister relationship of *Calamonastes* and *Camaroptera*, which had previously been inferred based on single-locus analyses only [37,38]. Johansson et al. [18] suggested *Eremomela* to be nested within Cisticolidae *,* contra Dickinson [20], who placed it in Phylloscopinae. However, they found contradicting evidence in their study: ODC1 and MB supported a close relationship with *Apalis*, while FGB placed *Eremomela* as sister to *Prinia* (no other cisticolids were included). Our combined analyses placed *Eremomela* with high support in the clade including *Apalis*.

The present study included six out of the eight genera and six out of the eleven species in the Malagasy endemic Bernieridae, and is the most complete analysis of this family to date with respect to number of loci, although one mitochondrial study included three additional species (one additional genus: *Cryptosylvicola*) [29], and one study based on MB, ODC1, LDH, GAPDH and MT-CYB also included the monotypic genus *Cryptosylvicola*[21]. All of the relationships inferred in the present study were strongly supported, except for the sister relationship between *Hartertula* and *Thamnornis*.

Clade 18 consisted of Aegithalidae and Cettiidae (including the genera *HyliaErythrocercus* and *Scotocerca*, which have been assigned to Cettiidae [1]). Alström et al. [22] noted that Cettiidae and *Scotocerca* shared certain morphological characters, such as 10 rectrices, whereas most passerines have 12. While *Erythrocercus* and *Scotocerca* were clearly related to Cettiidae *sensu* Alström et al. [14] in the present study, a close affiliation of *Hylia* to Cettiidae is questionable. *Hylia* has proved to be difficult to place before [23,24,26], although Beresford et al. [16] found strong support for an unresolved *Hylia*/ *Aegithalos*/ *Cettia* clade based on the nuclear RAG1 and RAG2. However, strong support was found for a sister relationship between *Hylia* and *Pholidornis* based on mitochondrial ND2 and 12S [26]. The latter relationship has previously been suggested based on anatomical details [39], and *Hylia* and *Pholidornis* have been placed in the family Hyliidae Bannerman, 1923 [26,39]. This seems a reasonable treatment, although it would be desirable to include both *Hylia* and *Pholidornis* in a multilocus analysis, preferably including additional loci compared to the present study.

With respect to *Scotocerca*, we suggest that it is better placed in a monotypic family rather than in Cettiidae. It is morphologically and ecologically highly divergent from the Cettiidae *sensu* Alström et al. [14] (which admittedly is in itself a morphologically exceptionally variable group; cf. [40]). Moreover, it is separated from Cettiidae *sensu* Alström et al. [14] by a long internode, both in the present study and in the one by Alström et al. [22]. We therefore propose a new family name:

### Scotocercidae, Fregin, Haase, Olsson and Alström, new family group name

Type genus *Scotocerca* Sundevall, 1872. Diagnosis: The genus *Scotocerca* includes a single polytypic species, *S. inquieta*, which is a small (c. 10 cm) warbler, with a long, slightly graduated tail with 10 feathers (outermost rectrices usually < 10 mm shorter than longest); three prominent rictal bristles; dark hair-like bristles on lower forehead, lores and chin; pale greyish or brownish upperside with some streaking, at least on crown; paler underparts, often more deeply coloured (buffish) on flanks, and usually with some streaking on breast; prominent pale supercilium and dark eye-stripe; rectrices rather dark, at least from below, usually with narrow pale tips (not on central pair). See del Hoyo et al. [41], pp. 465–466, and Plate 35, p. 462, and Alström et al. [22], Figure 2.

We also suggest that the genus *Erythrocercus*, which includes three species distributed in sub-Saharan Africa, be treated as a monotypic family rather than in Cettiidae. The same reasons as for *Scotocerca* apply, although *Erythrocercus* is even more different morphologically [22]. We therefore propose a new family name:

### Erythrocercidae, Fregin, Haase, Olsson and Alström, new family group name

Type genus *Erythrocercus* Hartlaub, 1857. Diagnosis: Small (c. 10–11 cm) flycatcher-like warblers, with prominent bristles around base of bill, moderately rounded tail with 12 rectrices; variously coloured and patterned plumages (mainly greenish above and yellow below in *E. holochlorus*; similar, but with a grey cap and rufous tail with dark subterminal band in *E. livingstonei*; and greyish upperparts with rufous cap and tail, and buffish throat/breast in *E. mccallii*). See del Hoyo et al. [41], pp. 327–328 and Plate 26, p. 324, and Alström et al. [22], Figure 2.

The family name Macrosphenidae for the sub-Saharan African “*Sphenoeacus*-group” of Beresford et al. [16] and Johansson et al. [18] is already widely used (e.g. [1]), but has not been formally described yet. Therefore, we here officially propose the name

### Macrosphenidae, Fregin, Haase, Olsson and Alström, new family group name

For the genera *MacrosphenusSphenoeacusMelocichlaAchaetopsSylvietta* and *Cryptillas*. Type genus *Macrosphenus* Cassin, 1859. Diagnosis: This family is defined based on monophyly (as found here and by Beresford et al. [16] and Johansson et al. [18]). The different genera are morphologically and ecologically highly divergent, with no known diagnostic morphological characters. The five species in *Macrosphenus* are 11–14.5 cm, with rather long, straight bills and (except in *M. kretschmeri*) rather short tails; plumage colours subdued, mostly various shades of dull greenish, yellowish, brownish and greyish; inhabits forest (see del Hoyo et al. [41], p. 641–642 and Plate 47, p. 640). Note that the position of *M. kretschmeri* in Pycnonotidae found by Alström et al. [14] was based on a misidentified specimen, as pointed out by Johansson et al. [42]. The single species in *SphenoeacusS. afer*, is 19–23 cm, with a long, strongly graduated, pointed tail; rufous cap, black malar stripe, and heavy streaking above and below; inhabits various grassy and scrubby areas (see del Hoyo et al. [41], p. 611 and Plate 443, p. 606). The single species in *MelocichlaM. mentalis*, is 18–20 cm, with a long, broad, rounded tail; uniformly brown above and paler below with contrastingly dark tail and black malar stripe; inhabits areas with grass and coarse herbage and forest clearings (see del Hoyo et al. [41], p. 611 and Plate 43, p. 606). The single species in the genus *AchaetopsA. pycnopygius*, is 16–17 cm, heavily streaked above and on breast, with rufous belly and flanks, distinct white supercilium and black malar stripe; inhabits rocky ground on hill sides (see del Hoyo et al. [43], p. 290–291 and Plate 24, p. 288). The genus *Sylvietta* contains nine species, which are small (8–12 cm) and extremely short-tailed; plumages various shades of grey, rufous, greenish and yellowish, no dark streaking; inhabit mainly forest (see del Hoyo et al. [41], p. 687–689 and Plate 53, p. 686). The single species in the genus *CryptillasC. victorini*, is 15–17 cm, with a fairly long, graduated tail, plain brown upperparts, plain pale rufous underparts, grey ear-coverts and pale orange iris; inhabits low, dense vegetation, often in moist areas (see del Hoyo et al. [41], p. 602 and Plate 42, p. 598). It was previously placed in the genus *Bradypterus*, but was shown to belong in this clade by Beresford et al. [16].

For names proposed after 1930, The International Code of Zoological Nomenclature [44] requires “a description or definition that states in words characters that are purported to differentiate the taxon” (Article 13.1.1), or “a bibliographic reference to such a published statement” (Article 13.1.2). As is evident from the above description of the family Macrosphenidae, it can be very problematic, or even impossible, to meet these requirements for family-group names (or genus-group names) that are defined based on clades in molecular-based phylogenies. In the case of Macrosphenidae, no diagnostic morphological characters that are shared by all its members are known, and in view of the enormous morphological diversity within this clade (which, at least in part, is likely to be shaped by the strongly divergent ecological adaptations among the genera), it is possible that no such characters will ever be found.

We have registered this publication in ZooBank under the following LSID: urn:lsid:zoobank.org:pub:DB5ADCC7-69D5-42AD-BCBE-B58BAC2C512A.

## Conclusions

The present study is the most comprehensive analysis of the superfamily Sylvioidea, both with respect to the number of taxa and the number of loci. The inferred tree is generally well resolved and well supported. However, several nodes deep in the tree remain uncertain, probably as a result of a rapid radiation of the families within Sylvioidea. All families except Cettiidae (*sensu* Gill and Donsker [1] but not *sensu* Alström et al. [14]) were strongly supported. Although the phylogenetic position of *Hylia* was uncertain, we tentatively support the recognition of the family Hyliidae Bannerman, 1923 for this genus and *Pholidornis*. We propose new family names for the genera *Scotocerca* and *Erythrocercus*, Scotocercidae and Erythrocercidae, respectively, and we formally propose the name Macrosphenidae, which has been in informal use for some time. We recommend that Paridae, Remizidae and Stenostiridae are not included in Sylvioidea.

## Methods

### Taxonomy

Taxonomy follows the IOC World Bird Names List Version 2.10 July 2011 [1].

### Taxon sampling and outgroup

We sampled 79 representatives of all 20 currently recognized families of the superfamily Sylvioidea (Table 1, Additional file 1), represented by up to ten genera per family. We also included three species with unreolved family affiliations: *Scotocerca inquieta*, *Erythrocercus mccallii,* and *Hylia prasina*.

The outgroup ( Additional file 1) consisted of the three corvoid species *Erpornis zantholeuca*, *Mystacornis crossleyi* and *Corvus corone,* with which the tree was rooted; a close relative of Passerida ( *Chaetops frenatus)*; two to three representatives from Passeroidea, Muscicapoidea, and Certhioidea; and representatives of Regulidae, Paridae, Remizidae and Stenostiridae.

New samples were collected according to the standards of the Swedish Board of Agriculture, although no formal application was required for this study.

### DNA extraction, amplification, sequencing and assembly

DNA was extracted according to Miller et al. [45] with slight modifications or using the QIAamp® DNA MiniKit (50) following the manufacturer’s protocol. The following loci were sequenced: the mitochondrial cytochrome *b* gene (MT-CYB; 1143 bp), the glyceraldehyde-3-phosphodehydrogenase intron 11 (GAPDH; 438 bp aligned), the complete nuclear lactate dehydrogenase intron 3 (LDHB; 624 bp aligned), the entire nuclear myoglobin intron 2 (MB; 765 bp aligned), the nuclear ornithine decarboxylase (ODC1) exon 6 (partial), intron 6, exon 7, intron 7 and exon 8 (partial) (in total 796 bp aligned), and a major part of the recombination-activating gene 1 (RAG1, 1934 bp). Not all loci were sequenced for all taxa ( Additional file 1). If fewer than two sequences were available for a family, this is indicated in Figure 2 for single-locus analyses. To reduce the risk of amplifying nuclear copies (numts) [46] in MT-CYB, this gene was amplified including flanking parts. PCRs were made up by single components or with Ready-To-Go PCR beads from GE Healthcare. PCR products were cleaned with ExoSap IT and products from cycle sequencing were cleaned with DyeEx 96Plate from Qiagen (only when the ABI sequencer was used). Sequencing was done on a LiCor DNA Sequencer Long READIR 4200 or on an ABI 3130xl Genetic Analyzer. Sequences were assembled manually in BioEdit [47] or with the Staden Package [48]. In addition, fibrinogen beta chain intron 5 sequences (FGB; 632 bp aligned) were retrieved from GenBank. GenBank accession numbers for all included sequences are given in the Additional file 1. Sampling localities and sample numbers are provided with the sequences in GenBank. Sampling procedures comply with the ARRIVE guidelines; no laboratory experiments were carried out, and no animals were injured during DNA sampling (blood samples taken in tarsal vein; complying with the Swedish Board of Agriculture’s ethical standards).

### Phylogenetic analysis

The sequences were aligned using MAFFT [49] with complementary manual adjustments. Base compositions of the four different genetic markers were tested for nucleotide bias using χ^2^ test of homogeneity across taxa implemented in PAUP* 4.0b10 [50]. All markers were tested for saturation effects with Dambe 5.2.34 [51,52]. Indices for substitution saturation were significantly smaller than the critical indices for each partition. Thus, saturation was no problem for the reconstruction of the phylogeny. Phylogenetic analyses were performed by Bayesian inference (BI) using MrBayes 3.1 [53,54] and maximum likelihood (ML) inferences were conducted with GARLI-PART 0.97 [55]. Nine data sets were analysed: all seven loci separately, all concatenated (complete dataset), and all six nuclear loci concatenated (nuclear dataset). Indels were treated as missing data in BI and ML. In both multilocus analyses, the data were partitioned by locus, using rate multipliers to allow different rates for the different partitions.

The data were also analysed in MrBayes 3.2 [53,54] in 14 partitions, with the coding sequences (MT-CYB, RAG1, exons of ODC1) partitioned by codon. A variable rate prior was applied to all partitions, which were unlinked using the “unlink” command. Instead of selecting a substitution model *a priori*, we used the “mixed” command to sample across the GTR model space in the MCMC analysis [56], with the addition of I + Γ to all partitions.

MrModeltest [57] was used in conjunction with PAUP* [51] to estimate the best-fit nucleotide substitution models for implementation in MrBayes, based on the Akaike Information Criterion (AIC; [58]) and AICc for smaller samples [59,60]. The proposed models were: GTR + I + Γ for MB-CYB, GTR + Γ for FGB, HKY + Γ for GAPDH, GTR + Γ for LDHB, HKY + Γ for MB, JC for the exons of ODC1, GTR + Γ for the introns of ODC1 and GTR + I + Γ for RAG1. As GARLI-PART can implement more models than MrBayes, for the ML analyses jModelTest [61] was used to estimate nucleotide substitution models, with the same criteria as for MrModeltest. The best-fit models were: TVM + I + Γ for MT-CYB, TPM2uf + Γ for FGB, HKY + Γ for GAPDH, TPM3uf + Γ for LDHB, TPM3uf + Γ for MB, JC for the exons of ODC1, GTR + Γ for the introns of ODC1 and TIM3 + I + Γ for RAG1. We conducted 100 ML search runs with GARLI-PART with random starting trees to obtain the tree with the maximum likelihood. Non-parametric bootstrapping was performed in GARLI-PART with 500 replicates for the combined, and 1000 replicates for single locus analyses. The resulting bootstrap trees were read into Treefinder version October 2008 [62,63] to obtain the bootstrap values, as GARLI-PART does not calculate consensus trees.

MrBayes was run with 4 to 8 chains for 10 to 21 million generation, in two parallel runs with default priors. In the single locus analyses of RAG1 temp = 0.1 was used, as with default priors no convergence of both runs was obtained, even after several runs up to 30 million generations. Convergence of parameters in BI was monitored using the program Tracer v. 1.4 [64]. Burnin was defined as those number of generations that were obtained before the average standard deviation of split frequencies remained below 0.01. Thus, consensus trees were calculated from 40000 to 160000 trees, combined from both runs. We regard nodes with maximum likelihood bootstrap values >85% as well supported, following Erixon et al. [65], as it corresponds roughly to a 0.95 probability that the analyses recovered a correct clade, and posterior probabilities (PP) > 0.95. Trees were edited using MrEnt [66].

## Competing interests

The authors declare that they have no competing interests.

## Authors’ contributions

SF carried out most of the sequencing, did the sequence alignment, statistical analysis and drafted the manuscript. PA, MH and UO participated in the design and coordination of the study, and the two former helped to draft the manuscript. PA and UO also participated in data acquisition. All authors read and approved the final manuscript.

## Supplementary Material

Additional file 1 **Samples used, with GenBank accession numbers.** DZUG = Department of Zoology, University of Gothenburg, Göteborg, Sweden; FMNH = Field Museum of Natural History, Chicago, USA; NRM = Swedish Museum of Natural History, Stockholm, Sweden; UCT = Percy FitzPatrick Institute of African Ornithology, University of Cape Town; UWBM = University of Washington, Burke Museum; VH = Vogelwarte Hiddensee, Zoological Institute and Museum, Ernst Moritz Arndt University of Greifswald, Greifswald, Germany; ZMUC = Zoological Museum of the University of Copenhagen, Copenhagen, Denmark. Sequences new to this study are given in bold. Click here for file
