# Supplementary material for: New insights into family relationships within the avian superfamily Sylvioidea (Passeriformes) based on seven molecular markers
Source: BMC Evol Biol. 2012 Aug 25;12:157. doi: 10.1186/1471-2148-12-157 (PMC3462691; doi:10.1186/1471-2148-12-157)
Supplement: Additional file 1 — Samples used, with GenBank accession numbers. DZUG = Department of Zoology, University of Gothenburg, Göteborg, Sweden; FMNH = Field Museum of Natural History, Chicago, USA; NRM = Swedish Museum of Natural History, Stockholm, Sweden; UCT = Percy FitzPatrick Institute of African Ornithology, University of Cape Town; UWBM = University of Washington, Burke Museum; VH = Vogelwarte Hiddensee, Zoological Institute and Museum, Ernst Moritz Arndt University of Greifswald, Greifswald, Germany; ZMUC = Zoological Museum of the University of Copenhagen, Copenhagen, Denmark. Sequences new to this study are given in bold. [file 1471-2148-12-157-S1.pdf]

Species	Locality	Sample numbers for new samples	GenBank No.						
			MT-CYB	FGB	GAPDH	LDHB	MB	ODC1	RAG1
**Ingroup**									
Abroscopus albogularis	China, Sichuan	DZUG:U1932	HQ706175	–	HQ706264	HQ706186	HQ706226	HQ706303	**JX236411**
Acrocephalus dumetorum	Russia, Yennessei		–	–	–	–	FJ883105	FJ883134	–
	Kazakhstan		–	–	–	FJ883062	–	–	–
	India, Punjab		–	EF626749	FJ357911	–	–	–	FJ358146
	Finland		AJ004773	–	–	–	–	–	–
Acrocephalus gracilirostris	Kenya	VH:B0529	AJ004782	–	–	FJ883063	FJ883095	FJ883135	**JX236412**
Aegithalos caudatus	Germany	VH:A1553 (B0668)	**JX236371**	–	–	**JX236296**	–	–	**JX236413**
	Sweden		–	EU680621	FJ357912	–	AY228281	EU680703	–
Alauda arvensis	Sweden		**JX236372**	EF626747	FJ357913	HQ333047	AY228284	EF625336	AY056978
Ammomanes deserti	Jordan	VH:A1592 (B0703)	**JX236373**	–	**JX236286**	**JX236297**	**JX236343**	–	**JX236414**
	Saudi Arabia	UCT #1 H. Harah 4/4/00	–	–	–	–	–	**JX236460**	–
Apalis flavida	South Africa	VH:B0745	HQ333036	–	HQ333097	HQ333049	HQ333069	HQ333083	**JX236415**
Arizelocichla masukuensis	Tanzania	ZMUC:117572 (O1783)	HQ333035	EF626698	–	HQ333048	**JX236344**	EF625287	**JX236416**
Artisornis metopias	Tanzania	ZMUC:118732 (O2943)	**JX236374**	EU686247	–	**JX236298**	**JX236345**	–	**JX236417**
	Tanzania	DZUG:3246	–	–	**JX236295**	–	–	**JX236461**	–
Atimastillas flavicollis	Congo		–	EF626721	–	–	–	EF625310	–
	West Africa	VH:A1575 (B0720)	**JX236375**	–	**JX236287**	**JX236299**	**JX236346**	–	**JX236418**
Bernieria madagascariensis	Madagascar	FMNH:431202	HQ333038	–	HQ333100	HQ333052	HQ333071	HQ333086	**JX236419**
Bradypterus baboecala	Kenya	VH:A0769 (B0706)	FJ883053	–	–	–	–	FJ883162	**JX236420**
	South Africa		–	–	HQ333098	FJ883090	DQ008525	–	–
Calamonastes simplex	Tanzania	ZMUC:O6645	**JX236376**	–	–	**JX236300**	**JX236347**	–	**JX236422**
Calamonastides gracilirostris	Kenya	VH:A0847 (B0960)	FJ883043	–	–	FJ883077	FJ883113	FJ883149	**JX236421**
Camaroptera brevicaudata	Senegal	VH:B0735	–	–	–	**JX236301**	–	–	–
	Rwanda	VH:B0027	**JX236377**	–	–	–	**JX236348**	–	**JX236423**
	Nigeria, Jos Plateau	DZUG 133	–	–	–	–	–	**JX236462**	–
Camaroptera chloronota	Uganda	ZMUC:O3270	**JX236378**	–	**JX236288**	**JX236302**	**JX236349**	**JX236463**	**JX236424**
	Uganda:Kibale	ZMUC:119031	–	–	–	–	–	–	–
Cettia cetti	Israel	VH:A1579 (B0732)	**JX236380**		–	HQ333053	–	–	**JX236425**
	France		–	–	HQ706263	–	HQ706225	HQ121555	–
Cettia diphone	Russia		–	–	HQ121536	–	–	–	–
	China, Shaanxi		DQ008510	–	–	–	–	–	–
	unknown		–	EU680634	–	–	EU680584	EU680714	–
Chamaea fasciata	USA, California		AJ534526	–	FJ357856	**JX236305**	FJ357927	FJ358025	FJ358091

Chrysomma sinense	China, Yunnan	VH:B0690	**JX236381**	–	FJ357857	**JX236306**	FJ357928	FJ358026	FJ358092
Cisticola carruthersi	Kenya	VH:A1585 (B0736)	**JX236382**	–	**JX236289**	**JX236307**	**JX236350**	–	**JX236426**
Crossleyia xanthophrys	Madagascar	FMNH:393280	HQ706177	–	HQ706269	HQ706191	HQ706231	HQ706309	**JX236427**
Cryptillas victorini	unknown		AY958231	EU680630	–	–	EU680602	EU680710	AY799815
Delichon urbicum	Spain		DQ008517	–	HQ333103	HQ333055	DQ008568	–	–
	unknown		–	EU680641	–	–	–	EU680721	–
Donacobius atricapillus	Paraguay		DQ008481	EU680643	FJ357915	HQ333054	DQ008533	EU680723	–
	unknown		–	–	–	–	–	–	AY319979
Dromaeocercus brunneus	Madagascar	FMNH:384749	HQ706160	EU680644	HQ706289	HQ706211	**JX236351**	EU680724	**JX236428**
Dumetia hyperythra	India, Mysore	VH:A0908 (B0741)	**JX236383**	–	FJ357859	**JX236309**	FJ357930	FJ358028	FJ358094
Eremomela gregalis	South Africa	VH:B0744	**JX236384**	–	–	**JX236310**	**JX236352**	–	**JX236429**
	unknown		–	EU680646	–	–	–	EU680726	–
Eremomela pusilla	Ivory coast	VH:A0766 (B0712)	–	–	–	**JX236311**	–	–	–
	Jos Plateau, Nigeria	DZUG:2825	**JX236385**	–	**JX236293**	–	**JX236353**	**JX236464**	–
Erythrocercus mccallii	Africa		AF096465	EU680647	HQ121544	–	EU680585	EU680727	–
Hartertula flavoviridis	Madagascar	FMNH:438721	HQ706131	EU680650	HQ706267	HQ706189	HQ706229	HQ706307	**JX236430**
Hippolais icterina	Sweden		DQ008479	–	HQ333104	–	–	–	–
	Ukraine, Crimea	VH:B0981	–	–	–	FJ883078	FJ883120	FJ883153	**JX236431**
	unknown		–	EU680651	–	–	–	–	–
Hirundo rustica	Germany	VH:A1574 (B0751)	**JX236387**	–	HQ333105	HQ333056	–	–	–
	Sweden		–	EF626748	–	–	AY064258	EF441240	–
	unknown		–	–	–	–	–	–	AY443290
Hylia prasina	Uganda		HQ333041	–	–	HQ333057	EU680583	EU680732	–
	Africa		–	EU680652	HQ121545	–	–	–	–
	unknown		–	–	–	–	–	–	AY319984
Hypergerus atriceps	Guinea	VH:A0865 (B0752)	**JX236388**	–	–	**JX236313**	**JX236355**	–	**JX236432**
	Nigeria, Jos Plateau	DZUG:2824	–	–	–	–	–	**JX236465**	–
Hypsipetes philippinus	Philippines	ZMUC:117589 (O1800)	**JX236389**	EF626742	–	**JX236314**	**JX236356**	EF625331	**JX236433**
Illadopsis puveli	Guinea	VH:A0762 (B0612)	**JX236390**	–	–	**JX236315**	**JX236357**	–	**JX236434**
	unknown		–	EU686236	–	–	–	–	–
Leptopoecile sophiae	Nepal	VH:B0762	**JX236391**	–	–	**JX236316**	–	–	**JX236435**
	China, Qinghai		–	EU680658	HQ706262	–	DQ008569	EU680738	–
Lioparus chrysotis	unknown	VH:A1576 (B0692)	**JX236393**	–	–	**JX236318**	**JX236359**	–	**JX236437**
	Vietnam		–	–	FJ357874	–	–	FJ358043	–
Locustella lanceolata	China, Hebei		HQ706139	–	HQ706275	–	HQ706235	HQ706313	–
	China, Beidaihe	VH:A1101 (B0761)	–	–	–	**JX236319**	–	–	**JX236438**
Locustella naevia	Sweden		HQ706147	–	HQ706282	–	HQ706242	HQ706320	EF568259
	Germany		–	–	–	**JX236320**	–	–	–

Macrosphenus flavicans	Congo		–	EF626751	–	–	EF625286	EF625340	–
	unknown		–	–	–	–	–	–	AY319987
Megalurus palustris	Philippines	ZMUC:02031	FJ883052	–	**JX236290**	FJ883089	–	FJ883161	**JX236439**
	India, Punjab		–	EU680661	–	–	DQ008529	–	–
Melocichla mentalis	Nigeria		DQ008500	–	HQ333107	–	DQ008551	HQ333090	–
	Ivory Coast		–	–	–	HQ333059	–	–	–
	Guinea	VH:A0866 (B0853)	–	–	–	–	–	–	**JX236440**
Mirafra javanica	Thailand		DQ008520	–	HQ333106	HQ333058	DQ008571	HQ333089	–
		NRM:20046819	–	–	–	–	–	–	**JX236441**
Nesillas typica	unknown		–	EU680665	–	–	EU680592	EU680744	–
Nicator chloris	West Africa	VH:A1439		–	–	**JX236323**	–	**JX236469**	–
	unknown		**JX236396**	EU680666	–	–	EU680603	–	AY319991
Orthotomus sutorius	Thailand		DQ008491	–	HQ333109	–	DQ008542	HQ333092	–
	India, Himachal Pradesh		–	–	–	HQ333061	–	–	–
	unknown		–	GQ242050	–	–	–	–	AY319992
Oxylabes madagascariensis	Madagascar	FMNH:438719	HQ706179	–	HQ706266	HQ706188	HQ706228	HQ706306	**JX236444**
Panurus biarmicus	Sweden		–	EU680668	FJ357919	–	FJ357983	FJ358083	FJ358150
	Austria	VH:A0855 (B0629)	**JX236397**	–	–	**JX236324**	–	–	–
Pellorneum capistratum	West Sumatra	VH:A0910 (B0915)	**JX236398**	–	–	**JX236326**	**JX236361**	–	**JX236445**
			–	–	–	–	–	–	–
Phyllanthus atripennis	Ivory Coast	VH:A1588 (B0883)	**JX236399**	–	–	**JX236327**	**JX236362**	–	**JX236446**
	Congo		–	–	FJ357888	–	–	FJ358057	–
Phyllastrephus cerviniventris	Malawi	VH:A1600 (B0891)	**JX236400**	EF626726	–	**JX236328**	**JX236363**	EF625315	**JX236447**
Phylloscopus collybita	unknown		Z73487	GQ242051	–	–	–	–	–
	Sweden		–	–	FJ357920	–	DQ125966	FJ358084	AY319997
Phylloscopus sindianus	Caucasus	VH:B0216	Z73478	–	–	HQ706224	–	–	**JX236448**
	Turkey		–	–	–	–	HQ706261	HQ706340	–
	Pakistan		–	–	HQ706302	–	–	–	–
Pnoepyga albiventer	China, Sichuan		HQ121521	–	FJ357889	–	FJ357959	FJ358058	FJ358124
Pnoepyga pusilla	China, Yunnan		–	–	FJ357890	–	FJ357960	FJ358059	FJ358125
	Sumatra	DZUG:3248	**JX236401**	–	–	–	–	–	–
Prinia bairdii	Cameroon		–	–	FJ357921	–	–	–	FJ358151
	unknown		AY352536	EU680675	–	–	–	–	–
	Tanzania	DZUG:475	–	–	–	–	**JX236364**	**JX236470**	–
Prinia familiaris	Indinesia, Java		DQ008490	–	HQ121547	HQ333063	DQ008541	HQ121557	–
Prinia gracilis	India, Punjab	DZUG:461	**JX236402**	–	–	–	–	**JX236471**	–
	Israel	VH:A0734 (B0632)	–	–	–	**JX236329**	**JX236365**	–	**JX236449**
Psaltriparus minimus	USA, Arizona		GU244418	–	–	–	–	–	–
	unknown		–	EU680678	–	–	EU680582	EU680757	AY319999

Pseudoalcippe abyssinica	Kenya	VH:B0863	AJ534548	–	–	**JX236330**	**JX236366**	–	**JX236450**
	unknown		–	EU680679	–	–	–	EU680758	–
Pycnonotus barbatus	Mauretania		HQ333043	–	HQ333110	HQ333062	HQ333075	HQ333093	–
	Cameroon		–	–	–	–	–	–	FJ358152
	Kenya		–	EF626746	–	–	–	–	–
Scotocerca inquieta	Tunisia		HQ333044	–	HQ333111	HQ333064	HQ333076	HQ333094	–
Seicercus tephrocephalus	Myanmar		HQ706182	–	HQ706301	HQ706223	HQ706260	HQ706339	–
Sinosuthora webbiana	China, Yunnan	VH:A0880 (B0619)	**JX236403**	–	–	**JX236333**	**JX236367**	–	**JX236452**
	unknown		–	EU680669	–	–	–	EU680748	–
Sphenoeacus afer	South Africa	VH:B0898	**JX236404**	–	–	HQ333066	**JX236368**	–	–
	unknown		–	EU680687	–	–	–	EU680766	AY799822
Spiloptila clamans	Mauretania	VH:A1598 (B0887)	DQ008495	–	–	**JX236335**	DQ008546	–	**JX236453**
	Mauretania	DZUG:3247	–	–	–	–	–	**JX236472**	–
Stachyris nigriceps	Vietnam		HQ333045	EU680688	–	HQ333065	–	HQ333095	–
	Thailand		–	–	FJ357900	–	FJ357969	–	FJ358135
Sylvia atricapilla	Germany		Z73494	EU680691	–	HQ333067	AY887727	–	–
	Sweden		–	–	EF441232	–	–	EF441254	EF568261
Sylvietta brachyura	Senegal	VH:B0902	**JX236406**	–	–	**JX236338**	–	–	**JX236454**
	Cameroon		–	–	–	–	DQ125960	–	–
Sylvietta whytii	Tanzania		DQ008501	EU680693	HQ121548	–	DQ008552	EU680772	–
Tesia castaneocoronata	India, Himachal Pradesh	VH:A0860 (B0683)	JN808933	–	**JX236294**	–	JN809081	JN809044	**JX236455**
			–	–	–	–	–	–	–
Thamnornis chloropetoides	Madagascar		HQ333046	EU680694	FJ357923	HQ333068	HQ333077	HQ333096	AY320004
Trochalopteron elliotii	China, Sichuan	ZMUC:121571(O5782)	**JX236407**	–	–	**JX236339**	**JX236369**	–	**JX236456**
			–	–	–	–	–	–	–
Turdoides squamiceps	Israel	VH:A1578 (B0906)	**JX236409**	–	**JX236292**	**JX236341**	**JX236370**	–	**JX236458**
			–	–	–	–	–	–	–
Xanthomixis apperti	Madagascar	FMNH:427370	HQ706181	–	HQ706265	HQ706187	HQ706227	HQ706305	**JX236459**
Yuhina flavicollis	unknown	VH:A1634 (B0843)	**JX236410**	–	–	**JX236342**	–	–	–
	Vietnam		–	–	FJ357908	–	FJ357977	FJ358077	FJ358143
Zosterops japonicus	China, Yunnan		–	–	FJ357910	–	FJ357979	FJ358079	FJ358145
	Korea		AB159168	–	–	–	–	–	–
**Outgroup**									
Catharus guttatus	USA,		EU619718	EU680632	–	–	DQ466820	EU680712	AY307184
Certhia familiaris	Sweden, Uppland		–	EU680633	–	–	DQ011861	EU680713	–
	unknown		–	–	–	–	–	–	AY056983
	Austria	VH:B0601	**JX236379**	–	–	**JX236303**	–	–	–
Chaetops frenatus	South Africa		–	–	–	**JX236304**	–	–	–

	unknown		AY228052	EU680635	EF441212	–	AY228289	EU680715	AY443266
Corvus corone	France		U86032	AY529982	FJ357914	–	FJ357980	–	–
	unknown		–	–	–	–	–	EU272116	AY056989
Culicicapa ceylonensis	Vietnam, Kon Tum Province	NRM:20036793	AF096453	EU680640	GQ369627	**JX236308**	EU680605	EU680720	AY443279
Erpornis zantholeuca	unknown	VH:A0907 (B0845)	**JX236386**	–	–	**JX236312**	**JX236354**	–	AY443339
Leucosticte nemoricola	Nepal	VH:A1651 (B0889)	**JX236392**	–	–	**JX236317**	**JX236358**	–	–
	Nepal	VH:A1652 (B0888)	–	–	–	–	–	–	**JX236436**
	Pakistan	DZUG:1552	–	–	–	–	–	**JX236466**	–
Montifringilla nivalis	Mongolia	VH:A1644 (B0873)	–	–	–	–	–	–	**JX236442**
	Mongolia	VH:A1645 (B0874)	**JX236394**	–	–	**JX236321**	–	–	–
	Russia	UWBM:75748	–	–	–	–	–	**JX236467**	–
	Germany		–	–	–	–	DQ244066	–	–
Mystacornis crossleyia	Madagascar	FMNH:345863	**JX236395**	FJ178360	**JX236291**	**JX236322**	**JX236360**	**JX236468**	**JX236443**
Parus major	unknown		EU167009	EU680670	–	–	–	EU680749	AY443314
	Sweden, Uppland		–	–	EU272098	**JX236325**	AY228310	–	–
Passer montanus	Sweden		–	EF626752	AY336586	–	AY228311	EU325847	–
	Pakistan		AY030118	–	–	–	–	–	–
	unknown		–	–	–	–	–	–	AF143738
Regulus regulus	Austria	VH:B0899	–	–	–	**JX236331**	–	–	**JX236451**
	unknown		AJ004762	–	–	–	–	–	–
	Sweden, Uppland		–	EU680682	–	–	DQ008572	EU680761	–
Remiz pendulinus	Sweden		AY228081	EU680683	–	**JX236332**	AY228319	EU680762	AY443328
Sitta europea	Sweden		AF378102	EU680686	–	**JX236334**	AY064257	EU680765	AY064272
Stenostira scita	South Africa		**JX236405**	EU680689	GQ369629	**JX236336**	EU680607	EU680768	–
	unknown		–	–	–	–	–	–	AY799823
Sturnus vulgaris	Sweden		HM633385	EU680690	–	**JX236337**	AY228322	EU680769	DQ466812
Troglodytes troglodytes	Austria	VH:B0900	**JX236408**	–	–	**JX236340**	–	–	**JX236457**
	Sweden		–	EU680696	–	–	AY228325	EU680775	–
